# Rapid Screening of Physiological Changes Associated With COVID-19 Using Soft-Wearables and Structured Activities: A Pilot Study

**DOI:** 10.1109/JTEHM.2021.3058841

**Published:** 2021-02-11

**Authors:** Luca Lonini, Nicholas Shawen, Olivia Botonis, Michael Fanton, Chandrasekaran Jayaraman, Chaithanya Krishna Mummidisetty, Sung Yul Shin, Claire Rushin, Sophia Jenz, Shuai Xu, John A. Rogers, Arun Jayaraman

**Affiliations:** Shirley Ryan AbilityLab 505538 Chicago IL 60611 USA; Department of Physical Medicine and RehabilitationFeinberg School of MedicineNorthwestern University Chicago IL 60611 USA; Department of Biomedical EngineeringMcCormick School of EngineeringNorthwestern University Chicago IL 60611 USA; Simpson Querrey Institute, Northwestern University Chicago IL 60611 USA

**Keywords:** COVID-19, diagnostics, digital health, soft electronics, wearable sensors

## Abstract

Objective: Controlling the spread of the COVID-19 pandemic largely depends on scaling up the testing infrastructure for identifying infected individuals. Consumer-grade wearables may present a solution to detect the presence of infections in the population, but the current paradigm requires collecting physiological data continuously and for long periods of time on each individual, which poses limitations in the context of rapid screening. Technology: Here, we propose a novel paradigm based on recording the physiological responses elicited by a short (~2 minutes) sequence of activities (i.e. “snapshot”), to detect symptoms associated with COVID-19. We employed a novel body-conforming soft wearable sensor placed on the suprasternal notch to capture data on physical activity, cardio-respiratory function, and cough sounds. Results: We performed a pilot study in a cohort of individuals (n=14) who tested positive for COVID-19 and detected altered heart rate, respiration rate and heart rate variability, relative to a group of healthy individuals (n=14) with no known exposure. Logistic regression classifiers were trained on individual and combined sets of physiological features (heartbeat and respiration dynamics, walking cadence, and cough frequency spectrum) at discriminating COVID-positive participants from the healthy group. Combining features yielded an AUC of 0.94 (95% CI=[0.92, 0.96]) using a leave-one-subject-out cross validation scheme. Conclusions and Clinical Impact: These results, although preliminary, suggest that a sensor-based snapshot paradigm may be a promising approach for non-invasive and repeatable testing to alert individuals that need further screening.

## I. Introduction

The COVID-19 pandemic is a global public health crisis, with over 50 million confirmed cases and more than 1.2 million deaths worldwide as of November 11^th^ 2020. Testing has continued to be a critical factor to control and reduce the spread of the disease by timely isolating and/or treating individuals who are suspected of infection [1]. With a proportion of asymptomatic infections estimated between 20% to 30% [2], [3], rapid testing for pre-symptomatic or asymptomatic patients could be key to ending the spread of COVID-19 [4].

Ongoing efforts are being directed at the development of novel rapid screening technologies [5], [6], but at present the primary method to test an individual for the presence of the virus is based on molecular testing, also known as RT-PCR (reverse transcription polymerase chain reaction), which detects the virus genetic material in a biological sample from the patient respiratory tract or saliva [7]. Although this is considered the most sensitive type of test, it has several drawbacks: for many testing facilities, test samples must be transported to a lab for analysis, creating a delay period into the diagnostic process that can range from a few hours to a few days or over a week. Further, infections that occur immediately prior to or following the test are not detected, and repeated testing is often not feasible due to limited resources. As a result, the current testing capacity, as well as delays in processing and delivering test results, remain a bottleneck that is limiting the effectiveness of public health containment measures [8].

In addition to molecular testing approaches, early indications of COVID-19 could be detected through changes in vital signs or other physiological characteristics. For example, increased resting heart rate and heart rate variability have been proposed as early predictors of illness [9]–[11]. Though unlikely to achieve the sensitivity or specificity of molecular testing, physiological monitoring could become a cost-effective and high-throughput method for first-pass screening of individuals at risk of COVID-19 infection, such as hospital staff, residents of long-term care facilities and essential workers. Repeated, proactive monitoring is crucial for such groups, and an approach based on easily measured physiological signals, beyond the common skin temperature checks currently in use in many public places, could help fill in the monitoring gaps between “gold standard” molecular tests.

Wearable sensors present an enticing avenue to detect physiological signals indicative of COVID-19 [12]. Detection of adverse events such as atrial fibrillation [13], Lyme Disease [14], stress [15], and even the spread of viral infections at the population level [10], proved to be possible through continuous, long-term monitoring of vital signs using consumer-grade wearables. Recent studies have adopted this paradigm for detecting the onset of COVID-19 infections, by recording changes in heart rate, physical activity, respiration and sleep data [16]–[19] over long periods of time. However, the logistics of a continuous monitoring approach, when applied on a broad scale, could become quite challenging [20]. Continuous monitoring requires one device per individual, and even when provided with a device, not all individuals will use it consistently [21]. Furthermore, the extremely large amount of data per person can create challenges for proper data management and processing at scale [22], [23].

Here, we discuss a different paradigm for detecting alterations in physiology due to COVID-19 using wearable sensors, based on recording physiological responses during a short sequence of activities, using a novel soft body-conforming wearable sensor that adheres to the throat. We present preliminary results of a larger trial and describe a proof of concept of how this paradigm could enable large-scale deployment of rapid testing to identify individuals at-risk who need further screening.

## II. Results

### A. “Snapshot” Detection of COVID-19

**FIGURE 1.**
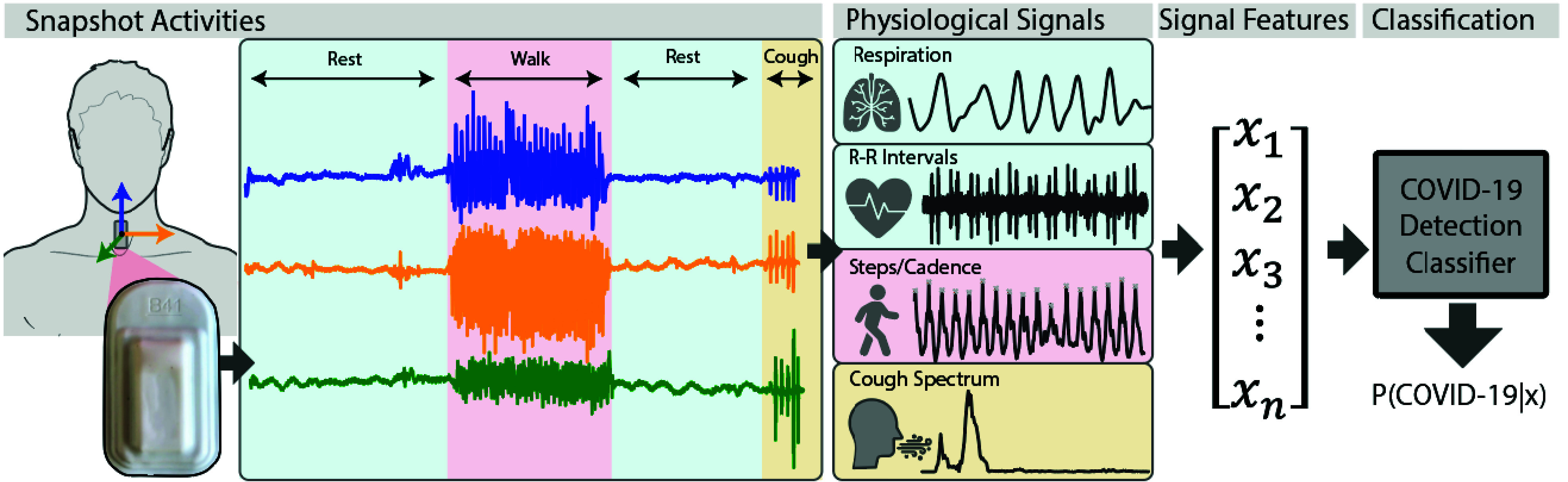
Accelerometer time series data were recorded using a soft wearable sensor [24] adhered to the suprasternal notch, as subjects performed a short set of predefined activities. Respiration and heartbeat dynamics, physical activity, and cough frequency information were derived from the recorded data. From these physiological signals, time and frequency domain features were extracted and fed into a symptom detection classifier trained to predict the presence of COVID-symptoms. The classifier outputs the probability of suspect symptoms based on the input signal features.

To address limitations in continuous physiological monitoring with wearable devices, we propose an alternative solution to detecting changes in physiology related to COVID-19 infections. Our method relies on two main components: a sensing platform capable of measuring physiologically relevant parameters, and a standardized sequence of activities (Fig. 1), which we refer to as a “snapshot”, designed to sensitively elicit responses indicative of a diseased state. By collecting a range of physiological signals during a snapshot, including heart activity, respiration, physical activity, and cough sounds, we hypothesized that changes due to COVID-19 could be detected.

The sensing device consists of a safe, soft, and reusable wearable sensor worn on the suprasternal notch and capable of recording mechano-acoustic signals through an embedded high-resolution accelerometer [24]. The device can measure broad body motions, such as those corresponding to walking, as well as subtle vibrations induced by sounds produced by heart beats, coughing, or breathing, thus making it possible to quantify physical effort and changes or anomalies in cardiac and respiratory physiology (see Section V for details). While our paradigm can be extended to other wearable platforms, the form factor of this device allows a more direct access to respiratory variables, including respiration dynamics.

### B. Pilot Study to Measure Physiological Trends From a Snapshot

Three different cohorts of individuals were outfitted with the soft wearable sensor to monitor physiological signals as they performed activities: Inpatient COVID-positive (n=10), Home-quarantining COVID-positive (n=5), and Healthy Controls (n=14). The Inpatient cohort consisted of individuals being treated at the Shirley Ryan AbilityLab, who had tested positive for COVID-19 and required physical rehabilitation resulting from severe COVID symptoms. The Home-quarantining cohort consisted of individuals who had milder symptoms and could recover from the infection at home. The Healthy Controls had no COVID-like symptoms or known exposure to the disease and were enrolled for an in-lab data collection. Demographics for the finalized set of participants are provided in Table 1 (see Section V for details). For statistical analyses, the Inpatient and Home-quarantining cohorts were combined into the COVID-positive group, while the healthy controls were labeled as COVID-negative.

**TABLE 1:** Patient Demographics. Comorbidity Abbreviations: HTN: Hypertension; DM: Diabetes Mellitus (type II); CA: Cancer; HF: Heart Failure; SLE: Systemic Lupus Erythematosus; HLD: Hypersensitivity Lung Disease

		Demographics				COVID-19 Related		Data Availability	
	Subject ID	Gender	Age	Height	Weight	Symptoms	Comorbidities	Instances	Days
			yrs	cm	kg			Pre/Post Gait/Cough	Pre/Post Gait/Cough
COVID+ Inpatients	SRAL2011F	F	78	153	50.3	Cough, fever	HTN, DM	2 / 4	1 / 3
	SRAL2012BM	M	55	180	78.9	SOB	HTN, asthma	2 / 5	2 / 3
	SRAL2014F	F	65	165	63	Cough, increased HR, fever, SOB	CA	4 / 17	2 / 10
	SRAL2019M	M	60	175	68	SOB	HTN, HF	3 / 5	2 / 3
	SRAL2020BF	F	64		97	SOB, cough	HTN, CA	4 / 4	1 / 2
	SRAL2024BM	M	54	184	139.6	Cough, COB, fevers	Obestiy	1 / 1	1 / 1
	SRAL2025F	F	53	165	82	SOB	SLE, HF, ECF 30%	4 / 6	2 / 4
	SRAL2032BM	M	52	187	80.9	Body pain, SOB	HTN	3 / 10	2 / 5
	SRAL2032F	F	59			Cough, SOB, Resp. FX,	HTN, HLD	11 / 5	6 / 4
	SRAL2033M	M	32	190	127.8	Cough, SOB, Resp. Fx	Obesity, HTN	3 / 1	3 / 1
COVID+ Home	SRAL-F-H1	F	31	170	70.3	Cough, mild symptoms	–	1 / 21	1 / 3
	SRAL-F-H4	F	21	150	47.6	Chest pain, minor cough, lost sense of smell and taste	–	3 / 1	1 / 1
	SRAL-M-H3	M	59	178	66.7	Fever, coughing, fatigue, body aches, chills	–	3 / 2	2 / 2
	SRALH8M	M	45	185	141.9	Fever	Obesity	1 / 1	1 / 2
Healthy Control	Control01M	M	37	172	70.3	–	–	1 / 2	1 / 1
	Control03F	F	32	162.6	63.5	–	–	1 / 2	1 / 1
	Control04F	M	33	165.1	56.7	–	–	1 / 2	1 / 1
	Control06F	F	43	172.7	79.38	–	–	1 / 2	1 / 1
	Control07M	M	42	180.3	77.6	–	–	1 / 2	1 / 1
	Control10F	F	22	160	59	–	–	1 / 2	1 / 1
	Control11M	M	36	170.2	79.4	–	–	1 / 2	1 / 1
	Control14F	F	23	152.4	47.6	–	–	1 / 2	1 / 1
	Control19M	M	31	180.3	81.7	–	–	1 / 2	1 / 1
	Control20M	M	24	170.2	68.0	–	–	1 / 2	1 / 1
	Control21M	M	32	172.7	74.8	–	–	1 / 2	1 / 1
	Control22M	M	34	182.9	98.9	–	–	1 / 2	1 / 1

**FIGURE 2.**
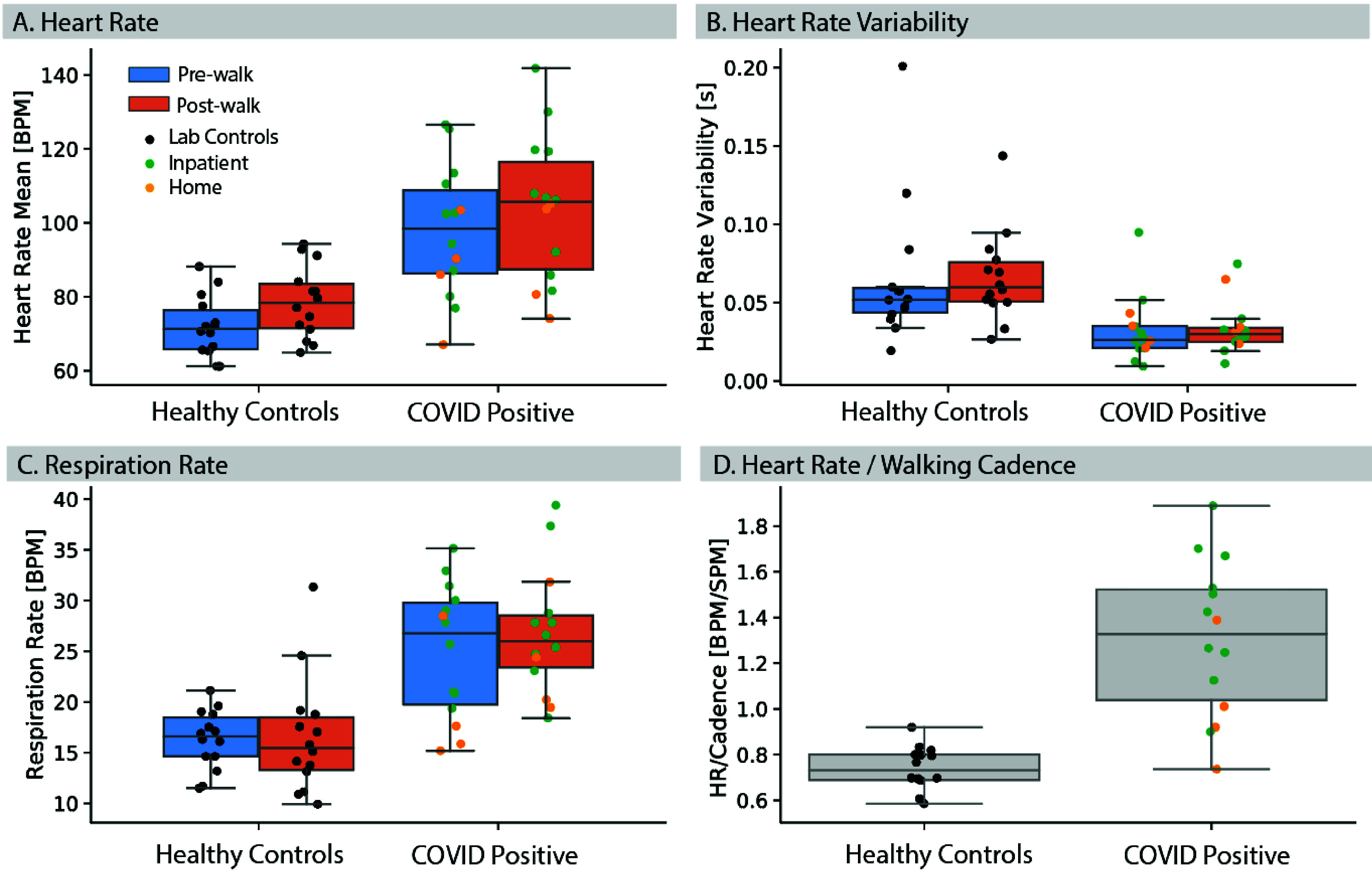
Pre-walk and post-walk physiological signals in Healthy Controls and individuals who tested positive to COVID-19, derived from accelerometer time series data. Participants who tested positive to COVID-19 (home-quarantined and inpatients) displayed altered heart rate (A), heart rate variability (B), respiration rates (C) and heart rate / walking cadence (beats per minute / steps per minute, (D)), compared to healthy controls.

For each subject, periods of rest, walking, and forced coughs were recorded using the soft wearable sensor attached to the suprasternal notch. We processed the accelerometer time series to derive physiological signals (see Section V) corresponding to respiration rates and R-R intervals during the resting phases, as well as walking cadence, for each activity snapshot (a sequence of rest, walk, rest). We compared the distributions of respiration rate, mean heart rate and heart rate variability (HRV, calculated as standard deviation of R-R intervals) prior to walking, as well as their changes before and after walking between COVID-positive and Healthy Controls (Fig. 2A-C). Acoustic features were extracted for the data collected during forced coughs.

Mean heart and respiration rates at baseline (pre-walk) were higher in participants who tested positive (**median HR-Healthy Controls: 71.4 beats per minute, COVID-Positive: 98.4 beats per minute** U=18.0, p<.001; **median Resp Rate**–**Healthy Controls: 16.6 breaths per minute**, **COVID-Positive: 26.8 breaths per minute**; U=26.0, p<.001), while heart rate variability (HRV) was lower (**median HRV Post– Healthy** Controls: 0.052 s, **COVID-Positive:** 0.026 s; U=28.0, p=.0012), relative to the control group. Therefore, we detected differences in individual physiological features between the 2 groups, which may be associated with a diseased state.

To understand the effect of exertion, we compared *within-group* changes (Post-walk vs. Pre-walk) of vital signs: the median heart rate in each group was higher following walking (**HR – Healthy Controls Post: 78.3 beats per minute, Pre: 71.4 beats per minute;** W=5.0, p=0.003; **COVID-Positive** **Post: 105.7 beats per minute, Pre:98.4 beats per minute**; W=7.0, p=0.004); neither Respiration Rate nor HRV changed significantly within each group as a result of walking (p>0.12). Pairwise *differences (Post-Pre)* in vital signs *between groups* were comparable, suggesting that the COVID-positive group did not show larger changes in any of the vital signs relative to the Healthy Controls as a result of exertion (**HR Post-Pre** **difference – Healthy** Controls: 6.7 beats per minute, **COVID-Positive: 5.7 beats per minute,** U=98.0, p=0.49; **Respiration Rate difference Post-Pre** – Healthy Controls: −0.43 bpm, **COVID-Positive**: **3.48 beats per minute;** U=69.0, p=0.095; **HRV Post-Pre** difference– Healthy Controls: 0.0027 s, COVID-Positive: 0.0037 s, U=95.0, p=0.45;). This could be due to several factors, including the fact that the inpatient group could have been already fatigued because of the physical therapy session.

We also examined the ratio of post-walking heart rate to walking cadence (Fig. 2D), as a further metric of cardiac response related to effort. We observed that individuals who tested positive had significantly higher values than controls (**Healthy Controls: 0.73 beats per minute/steps per minute; COVID-Positive: 1.33 beats per minute/steps per minute**; U=9.0, p<.001). Indeed, participants who tested positive tended to walk at a slower pace while having an increased heart rate after walking than the healthy control group (median **Cadence – Healthy**: **106.9 steps per minute**, IQR=[102.5, 111.1]; **Positive: 81.3 steps per minute**, IQR=[70.4,85.2]).

### C. Detecting Physiological Changes Due to COVID-19 From Snapshots

We also wanted to determine whether it is possible to detect a diseased state associated with COVID-19 from the physiological signals captured in a snapshot. Therefore, we trained a statistical learning model (Logistic Regression with Elastic Net regularization) on signal features derived from the R-R intervals, steps, respiration and frequency spectrum of cough signals. To evaluate the relative contribution of individual physiological features, we compared models trained on each individual physiological feature set (Pre/Post cardiac and cadence, Pre/Post respiration or cough) against one trained on the combined feature set (see Section V). The model was trained to classify the probability of COVID infection based on the label (COVID-positive vs. Healthy Control) of each snapshot.

We validated the model using a leave-one-subject-out cross validation, so to mimic the use-case of the paradigm [25], i.e. training on snapshots from a cohort with known diagnosis and testing on snapshots of a new participant with unknown diagnosis. Each COVID-positive participant had a variable number of data points (see Table 1), as cough and pre- and post-gait snapshots were recorded over multiple days of monitoring in the hospital or at home. Given the unbalanced number of datapoints between COVID-positive and Healthy participants, we randomly sampled one walk sequence and one cough sequence, with replacement, n=5 times for each individual to build a dataset for training and evaluating the model. This sampling was intended to simulate a brief screening data collection where each sequence was performed only once. This process was repeated 100 times to estimate confidence intervals on the model predictions, and ensured that each participant contributed the same weight to the model reported accuracy.

While combining different physiological features aided in separating the COVID-positive and negative groups (mean AUC **All** = 0.94, CI=[0.92, 0.96]), the improvement was marginal relative to a model trained using heart and walking cadence features only (AUC **HR**+**Cadence** = 0.93, CI=[0.91, 0.95]). Models trained on forced cough signals alone showed the lowest discriminative performance (AUC **Cough**=0.64, CI=[0.53,0.72]), suggesting that these features alone did not have sufficient discriminatory power in our cohorts. Whether this is due to a lack of resolution of the sensing device at capturing subtle changes in tracheal sounds, or the fact that these events were forced coughs from COVID-positive participants, and they were no longer in the acute phase of the disease, remains to be investigated in a future study.

Combining multiple physiological features also increased separability of the cohorts (Fig. 4). The model output probability, representing the probability of COVID infection for an individual in the test set, was overall higher for inpatients than for individuals quarantining at home, therefore suggesting that a model trained on an augmented feature set could help infer the likelihood of severe symptoms from mild ones (Fig. 4A-D).

**FIGURE 3.**
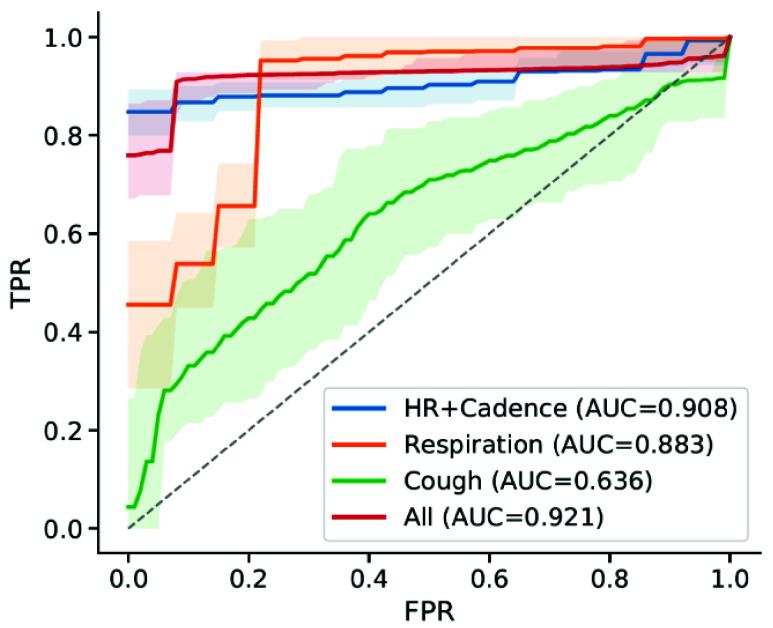
ROC curves for symptom detection models trained on different subsets of physiological features derived from the sensor data. Augmenting the set of physiological features aided detection of COVID-positive individuals. Mean ROC curves and AUC values shown are bootstrapped from for n=100 runs of the model. Shaded areas represent 95% confidence intervals.

**FIGURE 4.**
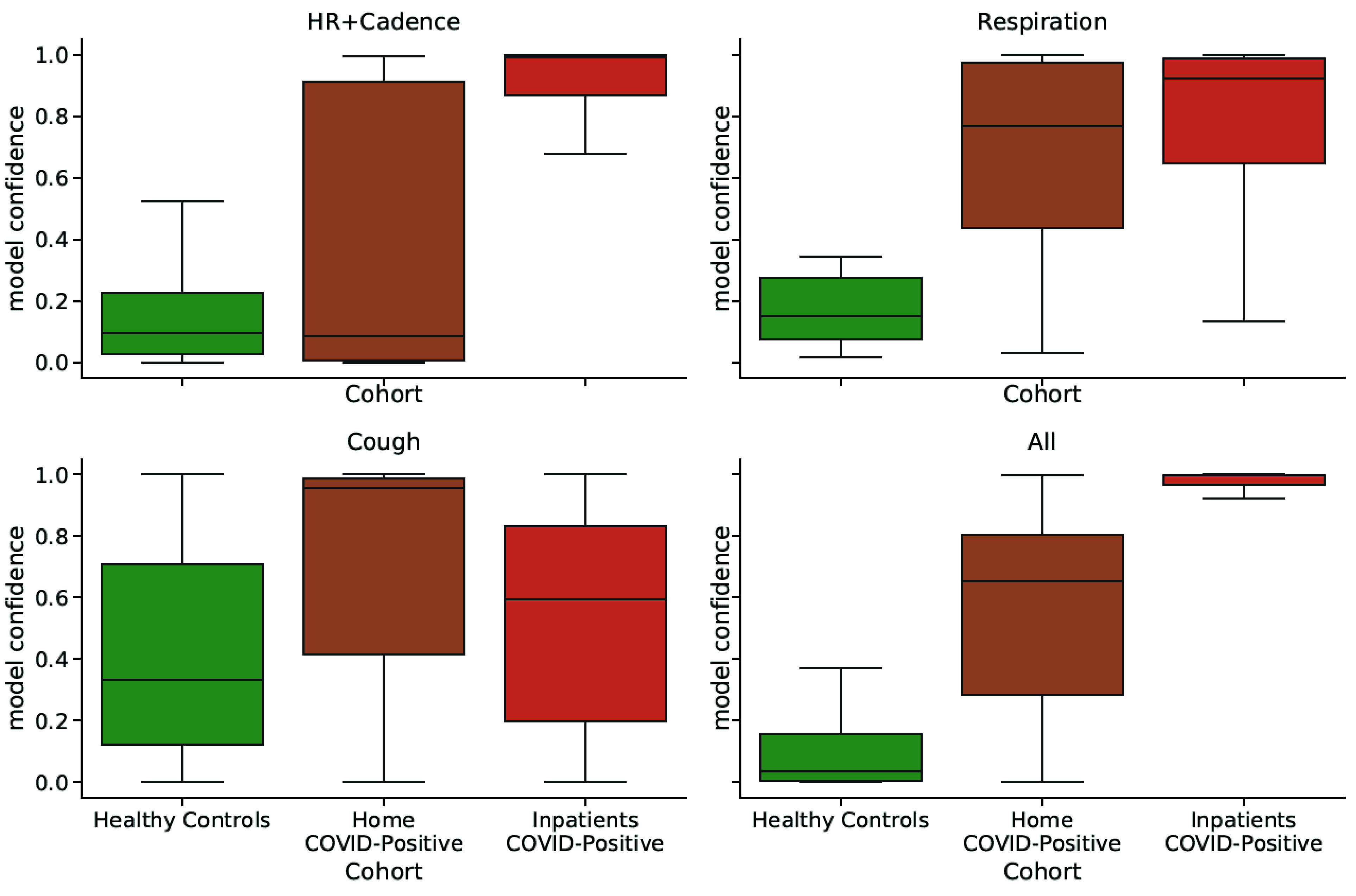
Distributions of model confidence values split by different participant cohorts. Combining features increased the separation of individuals by symptom severity: the output probability (confidence) for COVD detection was higher for individuals affected by severe conditions (inpatients) than for individuals quarantining at-home.

## III. Discussion

Skin-integrated sensors hold promise for continuous on-body sensing [26] which could be valuable for monitoring COVID-19 symptoms in an unobtrusive manner [27]. Here, we have shown that this technology could also be used to gather a snapshot of cardio-respiratory parameters, prior to and following physical effort, and determine whether an individual may need further screening. Using a chest-mounted soft accelerometer, we measured increased heart and respiratory rates and decreased HRV in individuals who had tested positive to COVID-19, relative to a Healthy Control group, while they performed a short set of standardized activities. This approach resembles stress tests that are commonly used in physical medicine to evaluate cardio-respiratory fitness [28]–[30]. However, we are not aware of any prior attempt of measuring a mild-stress-induced response to uncover changes in physiology of COVID-19.

The fact that alterations in physiological parameters were present in both inpatients with several existing co-morbidities and individuals quarantining at-home with no known underlying comorbidities suggests that the diseased state may have been the underlying cause of physiologically observed differences. Decreased time-domain measurements of HRV have been associated with a variety of conditions reflecting poor health [31], including inflammation and acute or chronic illness. Furthermore, we found that physical activity, cardiac, respiratory, and cough features gathered from a snapshot could be used to train a statistical learning model at discriminating individuals who tested positive in our sample.

Recent studies showed that crowdsourced data from smartphones or consumer-grade wearables on either respiratory, cardiac or cough sounds [17], [32]–[35] could potentially be used to develop biomarkers to predict the onset or detect the presence of COVID-19. A targeted snapshot of activities should exacerbate these physiological signs of Covid-19 to allow for more sensitive detection through wearable sensors. Further, fixing activities to a pre-defined sequence allows defining a precise context and facilitates the comparison of data across individuals, in contrast to the continuous sensing paradigm where data is gathered opportunistically.

A sensor-based “snapshot” approach measuring the physiological response to a physical stressor may provide additional prognostic information to detect COVID-19. Snapshot measures may also facilitate large-scale deployment of testing and be used to alert individuals that need further screening. With less data required to produce an evaluation of disease risk, early detection models could be more easily adapted and fine-tuned to specific populations, based on relatively small amounts of data and with reduced risk of statistical bias [36]. In addition to facilitating deployment, this paradigm may allow rapid collection of targeted data on diverse populations and provide insights into the manifestation of symptoms in these population, so to build a digital biomarker that fit different subsets of individuals.

While these results are encouraging, we need to acknowledge a number of limitations in our pilot study. First, our sample of Healthy Controls and COVID-positive groups is limited and not fully representative of the target population required to assess an early-screening methodology [37]. Therefore, the model presented is at risk of overfitting, and thus we are not yet able to quantify the actual sensitivity of this approach for detecting COVID-positive individuals. We also cannot ascertain whether the separation observed between the COVID-positive and the Healthy Controls group was uniquely caused by physiological changes induced by COVID-19 infections, or was attributed to other potential confounders, including co-morbidities existing in the inpatient cohort and age differences. At the time of the study, we were not able to enroll healthy age-matched individuals because of the significant risks posed by the pandemic in senior individuals, and thus were only able to run the trial on healthy individuals who were willing to participate and had a low risk of contracting the disease. As such, these factors could have inflated the accuracy of the model. Finally, the activities we selected here might not constitute the optimal set to uncover physiological changes of an ongoing COVID-19 infection. These factors limit the generalizability of our findings until a larger dataset more representative of the COVID-positive population is assembled. However, the main purpose of this study was not to create a generalizable statistical model, but rather to investigate whether physiological changes induced by COVID-19 could be detected from a snapshot activity sequence using our sensing device.

A key weakness of a snapshot paradigm is the lack of repeated measurements to assess changes in baseline physiological measures. However, the method can be extended to capture multiple snapshots over time, in order to monitor changes across days in a participant. Such an approach could eventually be used to measure the progression or regression of the respiratory infection. Similarly, in cases with a pre-specified target population (e.g. hospital employees or nursing home residents), this paradigm could easily be adapted to incorporate measurements taken at regular intervals in comparable circumstances, such as every other day at the end of the shift or after breakfast.

We are currently deploying a next-generation version of the sensing platform at multiple COVID-19 testing facilities, with the aim of collecting snapshots from a large cohort comprising thousands of individuals who may be in the early stage of the disease to obtain a reliable estimate of the sensitivity of this approach against RT-PCR. The new chest-mounted devices include an electrocardiogram (ECG), a temperature sensor, and an additional SpO2 finger sensor; these additional data will be used in conjunction with demographics and medical history to understand which activities and physiological features provide the highest diagnostic value in a snapshot approach. A more comprehensive set of snapshot activities is also being explored, which includes multiple periods of resting, walking, deep breathing, coughing, and breath-holding, to evaluate an optimal sequence of activities for eventual clinical use. The results of the ongoing multi-site trial will allow understanding the limits and potential use of this method for large-scale monitoring.

## IV. Conclusion

In conclusion, we showed that soft body-conforming wearable sensors could be used to capture an array of cardio-respiratory parameters during a short sequence of activities, which may help uncover physiological changes induced by respiratory diseases, such as COVID-19. While the results presented here are based on the specific sensor and activities we experimented with, the general approach presented is applicable to any type of wearable sensor capable of measuring relevant physiological signals. We hope that other researchers may benefit from exploring similar methods using different devices and activities, and help identify a more optimal approach to this “Snapshot” monitoring for COVID-19 symptoms.

## V. Methods and Procedures

All the participants provided written/verbal consent prior to their participation in this research study. Study procedures were approved by the Northwestern University Institutional Review Board (NU-IRB), Chicago, IL, USA (STU#00212522) on April 20, 2020. All study related procedures were carried in accordance with the standards listed in the Declaration of Helsinki, 1964.

### A. Patient Characteristics

During the first months of the pandemic, our hospital (the Shirley Ryan AbilityLab) received a limited number of participants who tested positive for COVID-19 and required physical rehabilitation as they recovered. Some of these individuals (n=14) provided informed consent to participate in our study and wear the sensor throughout the day, including during physical therapy sessions. In addition, we enrolled a group of 5 individuals who were recovering from the infection by quarantining at home. Both groups were asked to periodically perform specific activities: 5 deep breaths, 5 forced coughs, and 30 seconds of walking. Because of the severity of fatigue, inpatients with COVID-19 omitted the walking portion. These sequences were marked in the data via three taps on the sensor at the beginning and end and were intended as a reference set of activities while exploring the rest of the data.

In the course of our analysis of the data, we became interested in whether participants experiencing COVID-19 symptoms showed differences in vital signs, and whether these would be exacerbated by exertion. If so, it might be feasible to distinguish those with COVID from those without, based on small amounts of data. Because we were interested in resting vital signs both before and after exertion, segments of walking with accompanying pre- and post-walking rest were selected from the time series data for all participants. Isolation precautions for COVID positive participants allowed only trained nursing staff to interact with the individuals for sensor application. Thus, sensors were applied by nursing staff in the morning hours and were worn throughout the day (continuously) to ensure collection of gait during a given number of physical therapy sessions. As a reference for non-COVID physiological signals, several individuals (Healthy Controls, n=14) with no COVID-like symptoms or known exposure performed a sequence of activities that included 30 seconds of rest, 30 seconds of walking and 30 seconds of rest, in addition to the structured activities (cough, deep breathing) performed by COVID-positive participants. Participant demographics are provided in Table 1. Of the 19 individuals who tested positive, only n=14 had usable data, while the remaining ones were discarded due to data quality issues in the unmonitored data collection environment, such as loss of sensor skin contact or motion artifacts from talking. Although not logistically feasible for all of the COVID-positive subjects in this initial collection of data, directing each subject to perform the controlled sequence of activities without talking or unintended movements, as was done with the Healthy Control cohort in this study, would help reducing motion artifacts in future work.

### B. Sensing Device

The soft wearable wireless sensor used in this study was developed by the Rogers Research Group at Northwestern University. The device, worn on the suprasternal notch, was utilized to record the physiological signals of interest. In previous work, this device has been shown to be capable of measuring broad body motions, such as those corresponding to walking, as well as subtle vibrations induced by sounds produced by heart beats, coughing or breathing [24]. The device consisted of a high resolution 3-axis accelerometer embedded within a soft silicone package (Fig 1). The accelerometer x-axis (superior-inferior) and y-axis (lateral-medial) of the device sampled at 200 Hz. The accelerometer z-axis (anterior-posterior) sampled at 1600 Hz. The range of each accelerometer axis was ±2g. The sensing device also had a temperature sensor for continuous skin temperature recording, although this sensing modality was not used in this study, as a reliable reading of body temperature cannot be obtained through a skin-mounted sensor in the short context of our snapshot sequence due to environmental effects. The silicone sensor package adhered to the suprasternal notch of each subject using a disposable adhesive.

### C. Estimation of Respiration Rates

For each subject, the respiration rate during the pre-walk and post-walk resting periods were calculated using the accelerometer time series data. To compute the respiration rate, we used an approach based on reconstructing the angular motion induced by breathing by tracking the rotation of the gravity vector in the accelerometer signals [38]. This approach is briefly summarized here. The z-axis accelerometer signal was downsampled to 200 Hz to match the sampling frequency of the x-axis and y-axis. Each axis signal was filtered using a 2nd order Butterworth low-pass filter with a cutoff frequency of 1 Hz. The signal was normalized at each time point. The axis of rotation between two consecutive measurements of acceleration is calculated as follows:

\begin{equation*} r_{t}=a_{t}\times
                            a_{t-1}\end{equation*}

To reduce noise, each axis of rotation estimate was weighted by the angle change associated with each measurement, and the mean axis of rotation over a 5 second window length was computed using a Hamming window function. The current rotation angle, phi, was then computed as follows:

\begin{equation*} \phi _{t}=\sin ^{-1}((\bar
                            {a}_{t}\times \bar {r}_{t}) \cdot a_{t})\end{equation*}

**FIGURE 5.**
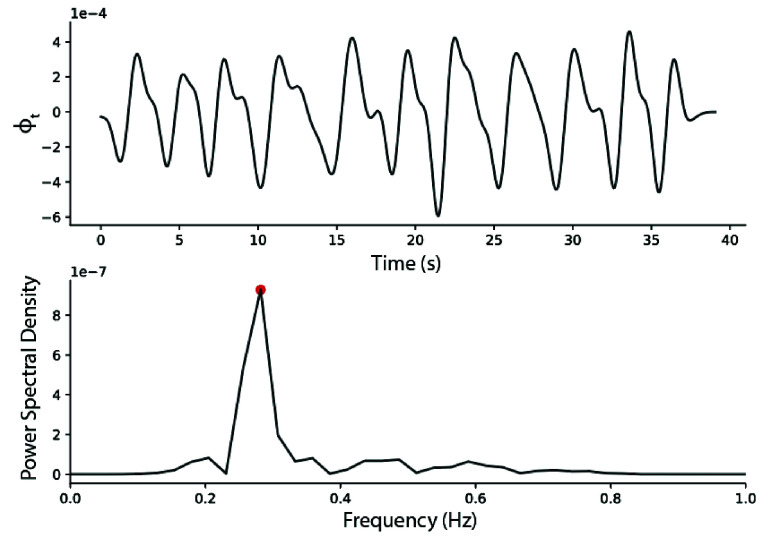
Example respiration signal taken from a Healthy Control patient. The peak corresponding to the respiration rate is highlighted in red.

To calculate the angular rate, $\phi _{t}$ was filtered with an 8^th^ order Butterworth band-pass filter with cutoff frequencies of 0.1 and 0.8 Hz, and then numerically differentiated with respect to time. The power spectral density of the angular rate was estimated using Welch’s method, and the respiration rate (breaths per minute) was taken to be the frequency at which the signal power is maximized (dominant frequency) (Fig. 5).

To quantify the regularity of the respiration rate, we also computed the number of peaks in the power spectrum, where a peak was identified as any spectral value equal or greater than 50% of the dominant frequency peak. These set of 4 features (Respiration Rate Pre- and Post-walk, number of FFT peaks Pre- and Post-walk) were input to the symptom detection model.

### D. Cough Signal Features

**FIGURE 6.**
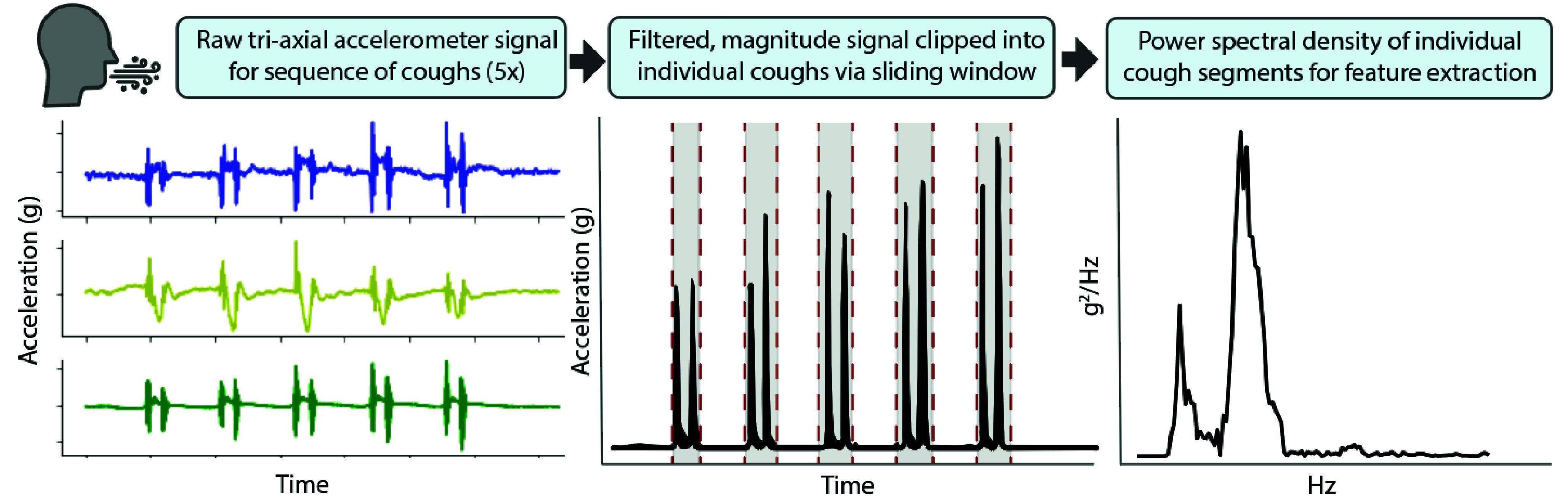
Signal processing of cough signals from an example Healthy Control subject.

Cough sequences performed and identified as five consecutive, voluntary coughs were manually clipped and extracted from the sequence of activities captured in a snapshot. For each sequence, x- and y- axes (200 Hz) were up-sampled to the frequency of the z-axis sampling rate (1600 Hz). A fifth-order, high-pass Butterworth filter (40 Hz) was applied to each axis and the vector magnitude of the acceleration signal was calculated. Cough sequences exceeding high-noise thresholds, based on percentage of zero-crossings with respect to the sequence duration, were discarded. Accepted sequence data was then input into a frequency-based sliding window cough detection function (window size of 0.2s, overlap of 50%). A wavelet denoising filter (5-level wavelet decomposition, sym5, universal thresholding rule, soft thresholding) was applied during this detection to eliminate high frequency noise prior to extracting power from the frequency domain of each sliding window. Those window regions identified with a power greater than a sequence-based threshold (25% of the sequence mean power) were designated as presence of cough (Fig. 6). Binary presence of cough was used to determine cough boundaries and clip data per individual cough. Finally, the following set of time and frequency domain features (21 total) were computed on each individual cough signal and averaged across the 5 coughs. These features were used as input to the symptom detection model; some of these features were derived from previous studies investigating classification of cough types from audio signals [39]:

**Time domain signal**: 1^st^-4^th^ Statistical Moments, Root Mean Square, Crest Factor, Duration, Maximum, Absolute difference, inter-quartile range, Sample Entropy, Lempel-Ziv complexity.
**Frequency domain (Power Spectrum)**: 1^st^-4^th^ Statistical Moments, Dominant frequency, Spectral Entropy, Spectral Centroid, Spectral Spread.

### E. Estimation of R-R Intervals

The R-R interval was extracted from sitting data that preceded and proceeded the walk bout. In order to extract the R-R interval from sensor data, a multi-tier signal filtering approach was adopted. First, the raw sensor data was de-trended and band pass filtered (2nd order Butterworth filter with cutoff frequency 0.3 Hz to 600 Hz) to remove any experimental noise and process noise. Following this, a Discrete Wavelet Transform (DWT) approach [40], at different characteristics scales, was used to filter the signal a second time. The characteristic scales were chosen such that the filtered output signal enhanced the energy of the signal and/or the peak of the signal for reliable identification of the peak R-R occurrences on the time series [41]. Following this, a threshold-based peak detection algorithm was used to extract the R-R intervals from the time series. Finally, the mean heart rate and heart rate variability (standard deviation of R-R intervals) were computed from the time series of R-R intervals.

### F. Estimation of Walking Cadence

To estimate the walking cadence, the walking portion of the sensor recording was manually extracted. Then the L2-norm of the acceleration was computed, and the stepping frequency (cadence) was computed as the dominant frequency of the FFT of this signal.

### G. Classification Model and Statistical Analysis

Statistical comparisons of physiological trends were performed with non-parametric statistical tests (Wilcoxon Signed-Rank Test and Mann-Whitney U Test) to account for the non-normality of distributions and the small sample size. P-values were corrected for multiple comparisons using Bonferroni correction. We used a correction factor of 9 ( $3 \times 3$ comparisons) which yielded a corrected p-value of 0.0056.

**FIGURE 7.**
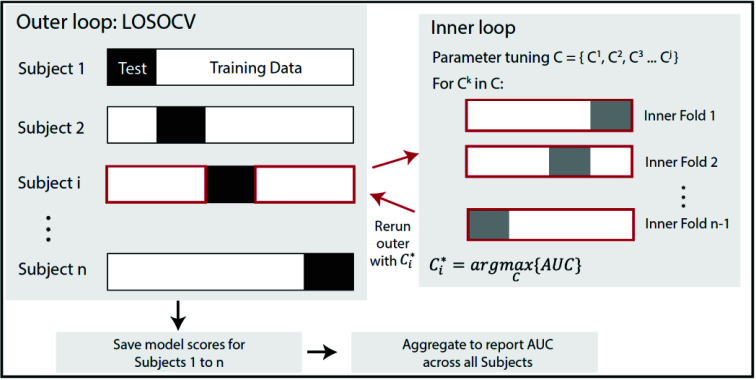
Hyperparameter optimization using nested cross validation. A leave-one-subject-out cross validation outer loop is utilized to report model performance (Area Under the Curve or AUC), where a single subject i is used as the test set, and the remaining subjects as the training set. For each subject i in the outer loop, an inner loop is run on the corresponding training data to find an optimal value of the regularization parameter. This optimal parameter is then used to re-run the outer loop and the process repeated until each subject has an optimal parameter value.

We trained a regularized logistic regression (elastic net [42]) model to detect the presence of COVID-like symptoms based on the physiological signal features. The model was implemented using the Scikit-learn library 0.23.2 in Python 3.7.6. The total number of data points (available walking and cough snapshots) across all participants was 288. In each of the n=100 bootstrap runs, we sampled with replacement n=5 snapshots from each participant, for a total of 135 samples. The full model combining all the features used a total of 30 input features. We performed a grid search to optimize the regularization hyperparameter $C$ for each set of features independently. To limit overfitting, we employed a nested cross validation (i.e. nested loop within the leave-one-subject-out cross validation used to report model performance), as shown in Figure 7. The ratio of L1 to L2 regularization was set to 0.5. The feature selection processes resulting from the elastic net regularization, indicated that heart rate, HRV, respiration features and walking cadence all had similar importance (i.e. coefficients) in the final model. Amongst cough features, spectral spread and IQR had the highest coefficients in the trained model, while the remaining cough features had relatively low contribution.
